# A large-scale dataset for training deep learning segmentation and tracking of extreme weather

**DOI:** 10.1038/s41597-025-05480-0

**Published:** 2025-07-05

**Authors:** Sol Kim, Andre Graubner, Lukas Kapp-Schwoerer, Karthik Kashinath, Konrad Schindler

**Affiliations:** 1 University of Chicago, Urban Theory Lab, Chicago, 60615 USA; 2 ETH Zurich, Institute of Geodesy and Photogrammetry, Zurich, Switzerland; 3 NVIDIA Corporation, HPC + AI, Santa Clara, 95051 USA

**Keywords:** Atmospheric science, Natural hazards

## Abstract

As Earth’s climate continues to undergo changes, it is imperative to gain understanding of how high-impact, extreme weather events will change. Researchers are increasingly relying on data-driven, learning-based approaches for the detection and tracking of extreme weather events. While several attempts to generate datasets of hand-labeled weather or climate have been made, a significant challenge has been to gather a sufficient number of expert-annotated samples. To address this challenge, we introduce the largest dataset of expert-guided, hand-labeled segmentation masks of extreme weather events. It contains global annotations for atmospheric rivers, tropical cyclones, and atmospheric blocking events from the European Centre for Medium-Range Weather Forecasting’s reanalysis version 5. Every timestep for each event is annotated by two separate annotators to bring the total number of labeled timesteps to 49,184. Professional annotators were trained and guided to identify these features by domain-experts, and event-specific experts were consulted for each of the annotation guides. The resulting annotations are demonstrated to have characteristics similar to other methods and those generated directly by domain experts.

## Background & Summary

It is unequivocal that, due to human activities, the Earth’s climate is changing and will continue to undergo extensive changes for the foreseeable future^1^. An important aspect of this process are changes in the occurrence of weather extremes such as heatwaves, coldsnaps, heavy precipitation, and tropical cyclones^2^. Thus, it is imperative to gain better understanding of how high-impact, extreme weather events will shift. The detection and tracking of these events is at present still largely reliant on human-engineered heuristics that are known to be fraught with various inconsistencies and deficiencies^3–7^. For example, many detection algorithms exist for each event type and different heuristic rules have led to large discrepancies in even the most basic statistics regarding extreme weather events, i.e., their frequency counts or distributions^5–8^. A necessary requirement to accurately characterize extreme weather events in large datasets is to be able to detect and track them in a reliable and repeatable manner.

To address this challenge, we have created *ClimateNetLarge* - a dataset of dense annotations of extreme weather events, which can either be analyzed on its own or serve as a basis for data-driven approaches, including in particular deep learning (DL). Data-driven approaches can learn to recognize target patterns based on a diverse set of annotations, thus removing the need for custom, threshold-based heuristics. Similar efforts have previously been made to collect hand-annotated labels from weather and climate experts who can use domain expertise to ensure quality and resolve edge cases and errors that algorithms may miss. However, doing so requires coordinated labeling campaigns^9^, which are costly, time-consuming and logistically challenging.

Our target was to collect a large-scale dataset of hand-labeled segmentation masks for atmospheric rivers (ARs), tropical cyclones (TCs), and blocking events. These events are associated with extremes in precipitation, wind, temperature, and drought - aspects that are critical to consider under a changing climate^10^. To that end, we turn to professional crowd-labeling, guided by expert instructions and training. In total, we have generated 49,184 hand-labeled timesteps, an order of magnitude more than previous annotated datasets of extreme weather events^9^.

The selected weather events share two qualities: they are responsible for catastrophic socioeconomic impacts^11–14^ and they are notorious for their complex characteristics; many different detection algorithms have been produced for these events which rely on different thresholds, variables, and/or geometries^5,6,8^, making them difficult to map reliably at the global scale with heuristic detection rules. ARs are a global phenomena transporting moisture and energy through mid-latitude regions and reaching into polar latitudes. These features are associated with numerous hazards such as floods, high winds, and debris flows among other less commonly studied impacts^15^. In the western U.S., where most of the AR literature has historically focused on, ARs are responsible for about one billion dollars of flood-related damage per year^13^. In other regions, such as western Europe, western South America, and Australia/New Zealand, AR events have also been linked to flooding and their absence has been linked to droughts^15,16^. One study found that approximately 300 million people across the global are exposed to flood risk due to the occurrence of ARs with the most significant regions found in California, the Mississippi basin, in the Parana River basin, in the Iberian Peninsula, southern Iran, the Amur and Yangtze Rivers, and the Murray-Darling basins; this highlights the large regional scope of AR impacts^16^. In a review presented by the World Meteorological Organization (WMO) Tropical Cyclone Programme, TCs have been responsible, on average, for 43 deaths and 78 million dollars of losses everyday for the past 50 years meaning TCs are responsible for one third of both deaths and economic losses from weather-,climate and water-related disasters globally^17^. Blocking events are associated with devastating heat waves, coldsnaps, and extreme precipitation events^12^. For example, blocking has been associated with the European heatwave in 2003 which claimed 30,000 lives due to unrelenting heat^18^ and extreme precipitation events over the continents of Asia, North America, and Europe^19^.

## Methods

**Fig. 1:**
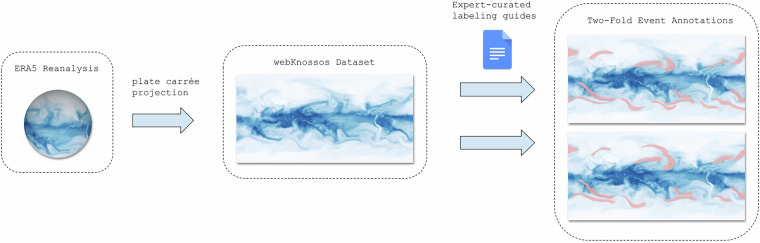
Schematic of the labeling workflow. After pre-processing the ERA5 reanalysis data, multiple crowd-labelers annotate each timestep according to expert-curated labeling guides. Shown here is the total column water vapor variable and only the corresponding atmospheric river annotations although more variables were available to annotators and all event types had two annotators label each timestep.

The following section provides an overview of the data and methods used to generate our dataset of segmentation masks for ARs, TCs, and blocking events as seen in Fig. 1. We describe the reanalysis data set used, preparation of the data for use within the labeling tool, the labeling process, and finally, the packaging of the final dataset.

### Reanalysis Data

Our dataset is derived from the global European Centre for Medium-Range Weather Forecasts Reanalysis version 5 (ERA5)^20^, which is publicly available through the Copernicus Climate Data Store website. Several variables were selected at hourly timesteps (for ARs and TCs) or daily timesteps (for blocking events). For ARs and TCs, professional hand-labelers were provided with global images of total column water vapor (TCWV), vertically integrated water vapor transport (IVT), and mean sea level pressure (MSLP). For blocking events, we provided global images of the 500 hPa geopotential height anomaly. These variables are relevant to typical definitions of the respective features. For ARs and TCs, we have selected 9,850 timesteps between 1980-2022, sampled uniformly (each timestep has an equal chance of being chosen) at random without replacement (a timestep may not be chosen twice), in order to cover a variety of climate conditions, seasons, and times of day. For blocking events, we use daily, consecutive timesteps that span the period from 2000 to 2013. Due to the predominantly temporal nature of blocking events, we chose not to randomly sample timesteps but to rather provide a continuous record, as the event duration is important for most studies. We have abstained from utilizing ERA5 data prior to 1980 to avoid biases inherited from pre-satellite observation technology.

### Labeling Tool and Crowd-sourcing Annotators

**Fig. 2:**
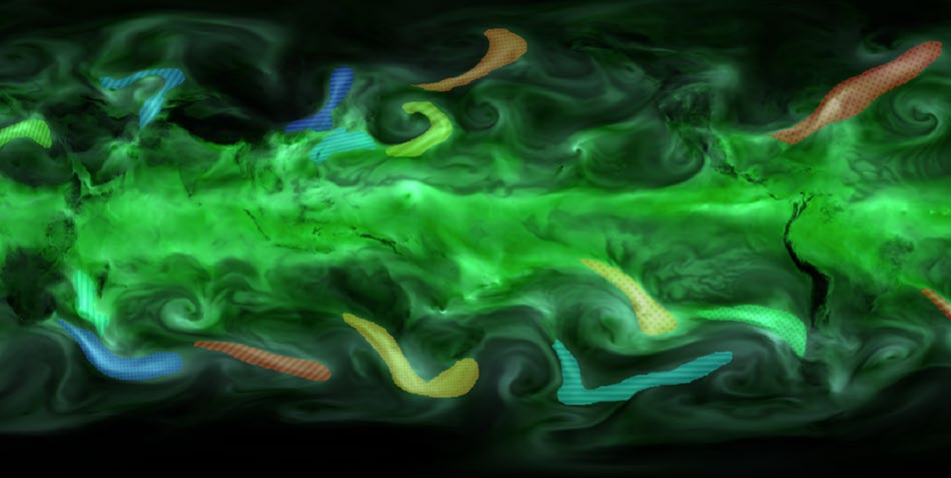
The webKnossos interface used for labeling displaying the total column water vapor field in green. The colored and patterned polygons show examples of one annotator’s atmospheric river labels.

Running consistent labeling campaigns for segmentation at scale is challenging. This issue is exacerbated by the specialized domain knowledge needed for atmospheric data: average crowd-labelers are not familiar with weather extremes like atmospheric rivers or blocking events, and common annotation tools do not support the kind of multi-channel data needed to annotate them. We overcome these difficulties by employing webKnossos^21^, a company originally focused on crowd-sourcing the creation of datasets for bio-medical image segmentation. Their suite of online tools is by design able to display the kind of complex, multi-channel data that annotators need to inspect for our task, and also provides a fine-grained system to track progress, update annotator instructions based on expert feedback, and identify potential quality issues. webKnossos provides a python package to process data arrays in python and both prepare them in a format that works with the tool and directly uploads them for use on the online webKnossos interface (https://docs.webknossos.org/webknossos-py/)^21^. This package was used to process the appropriate ERA5 variables for each event type; more details are in the Data Packaging section. Through the webKnossos interface, each variable is stored as a separate channel with functionality able to show a single channel or combinations of channels at the same time; thus, each timestep contains multiple channels, or variables, for the annotators to inspect. Each variable is also scalable to appropriate units for annotation purposes. For example, IVT can be scaled to highlight minimum values of around 100 kg/m/s and maximum values of around 750 kg/m/s to allow annotators to easily locate regions of high IVT; a feature relevant especially relevant for AR identification. Fig. 2 shows an example of polygons drawn through the webKnossos interface to denote ARs, overlaid on a corresponding image of TCWV. webKnossos employs professional annotators and are thus able to generate large volumes of labels that would be difficult to source from climate and weather experts alone, given the sheer amount of man-hours required and the one-off effort each new annotator must spend to familiarize themselves with the labeling tool. Depending on the individual annotator and how comfortable they were for a given type of event, labeling a single timestep for one event type took anywhere from less than a minute to over ten minutes.

### Labeling Guides

**Fig. 3:**
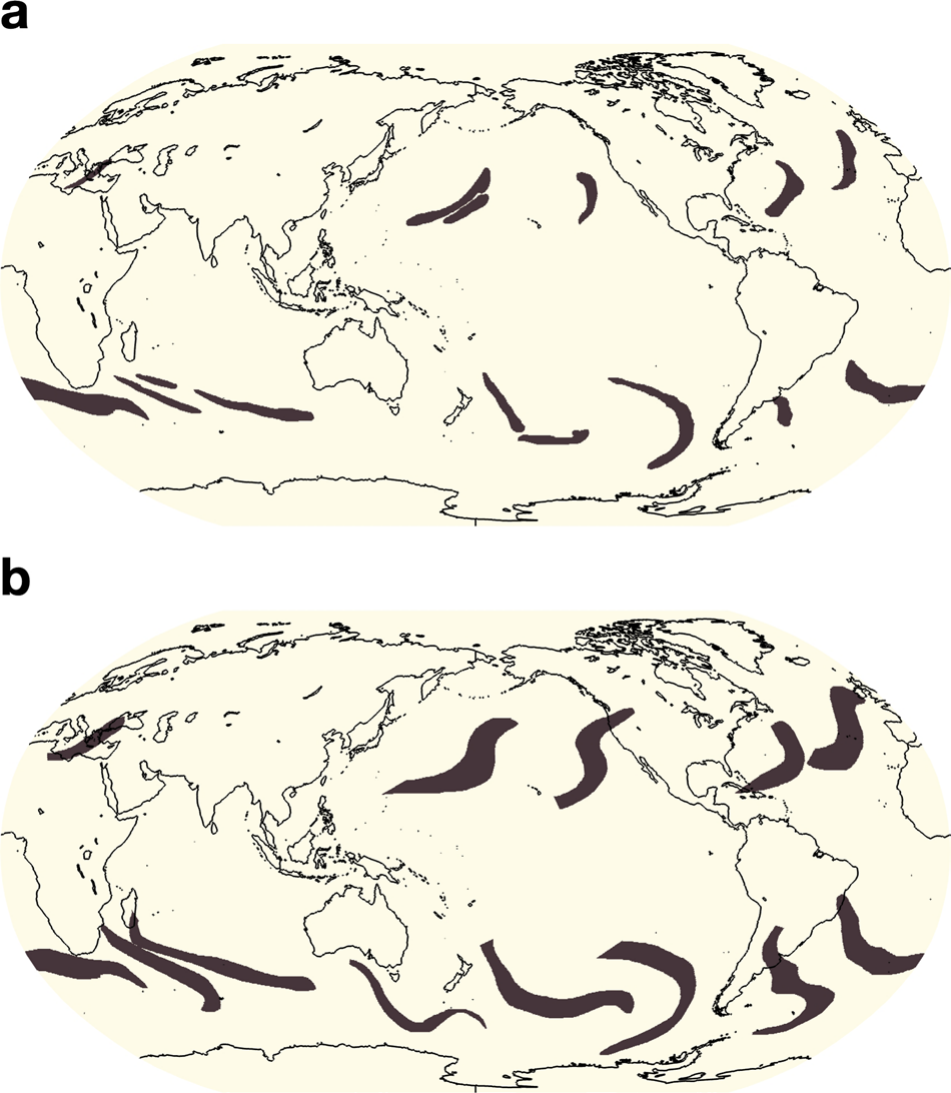
(**a**) and (**b**) show two different annotators’ labels of atmospheric rivers for the same timestep. Annotator (**a**) tends to draw smaller boundaries and distinguishes more atmospheric rivers whereas annotator (**b**) tends to draw larger boundaries and distinguishes less features. To note, both annotators agree on all areas of atmospheric rivers aside from annotator (**b**) finding one extra atmospheric river south of Australia.

To instruct the crowd-labelers we created labeling guides, based on input and feedback from event-specific climate experts. These guides provide *(i)* background information on the nature of the specific extreme weather event, *(ii)* concrete instructions for annotation workflows that specifically utilize the webKnossos tooling, and *(iii)* positive, negative, and edge-case examples. To further improve the dataset, we updated the guides iteratively in response to quality control of the incoming labels and to questions from the annotators that arose during the annotation process. This close dialogue with the crowd-labelers was crucial to ensure labelers had acquired the necessary understanding of each event type. While it was important to ensure that the annotators had a strong understanding of how to identify and delineate these extreme weather event types, we took care not to provide overbearing instructions so as to preserve diversity within the reasonable definition uncertainty and avoid steering all annotators toward a specific labeling bias. For example, Fig. 3 and 7 show how two different annotators labeled ARs and TCs in the same timestep. In this particular case, the annotators exhibit agreement over the locations of major ARs (with one exception off the southern coast of Australia), but to some level disagreed about the boundaries of these features; annotator **a** tended towards tighter and narrower masks, including nearby but separate ARs; whereas annotator **b** drew larger, more relaxed boundaries and merged nearby ARs into one mask.

In Fig. 2, polygons with varying color and/or texture denote different ARs. Each event is associated with a unique event ID, hence the separate color and/or texture per event. For readability we display only TCWV in the background, but note that during annotation, crowd-labelers viewed multiple data channels (depending on the event type). For details about the data available and our recommendations on utilizing each data field, we refer readers to the annotation guides. We briefly summarize some of the guidance provided for each of the event types. For ARs, annotators were to *(i)* primarily rely on zones of enhanced TCWV and IVT, *(ii)* look for long, narrow columns (at least a 2:1 ratio of length versus width) from the tropics/subtropics oriented towards the poles, *(iii)* avoid areas along the equator and cyclonic features, and *(iv)* label roughly 6-12 AR events globally for each timestep and no timesteps should be without any ARs^8,15^. For TCs, annotators were to *(i)* use MSL to identify low pressure anomaly zones, *(ii)* rely on TCWV and IVT to find concentrated, spiraling features associated with those low pressure zones, and *(iii)* generally focus on the tropics/subtropics for labeling^9,22,23^. For blocking events, annotators were to *(i)* use the 500 hPa geopotential height anomaly field focusing strictly on positive anomalies, *(ii)* look for nearly stationary, positive anomalies at roughly country size scales, and *(iii)* and focus on latitudes between 25-75° N and S respectively^6^. The labeling guides can be viewed at the following web links: AR Guide, TC Guide, Blocking Guide.

### Data Packaging

ERA5 reanalysis data are available on a regular latitude-longitude grid at 0.25° × 0.25° resolution. To process the atmospheric features for the webKnossos annotators, we load the NetCDF^24^ ERA5 data into python arrays and project the data onto a flat plane using a *plate carrée* equirectangular projection (relying on the NetCDF regular latitude-longitude grid) through the webKnossos python package (https://docs.webknossos.org/webknossos-py/)^21^. This package saves the resulting data array and image to the webKnossos online system. The processing script is made available (see section Code Availability).

After the annotation process, we package the labels into easily accessible files in NetCDF format^24^. Each event type is packaged separately in single timesteps for ARs and TCs, and series of 10 consecutive days for blocking events (see Fig. 12). More details on the specific structure of files are provided in the Data Records section.

### Data Overview

**Fig. 4:**
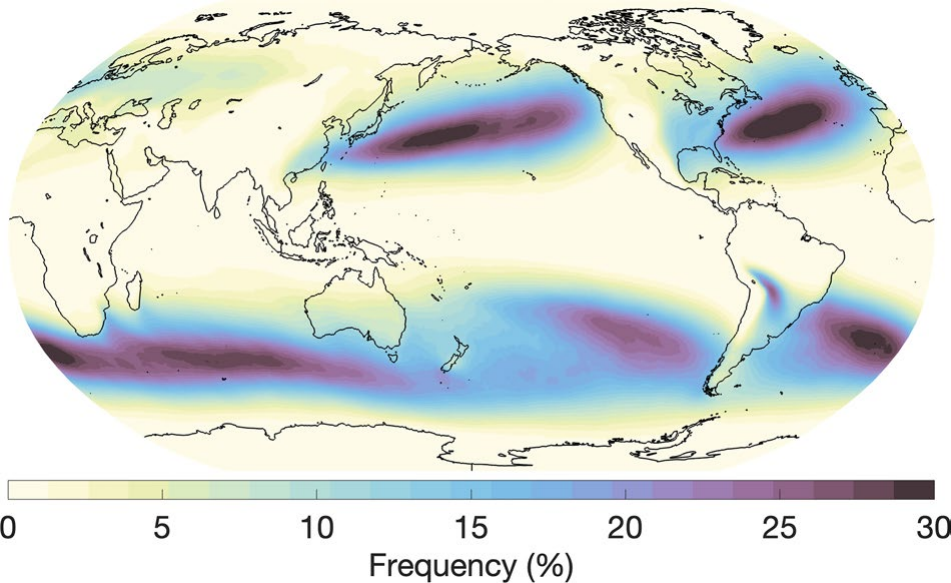
Annual global atmospheric river frequencies based on all annotated timesteps and all annotators.

**Fig. 5:**
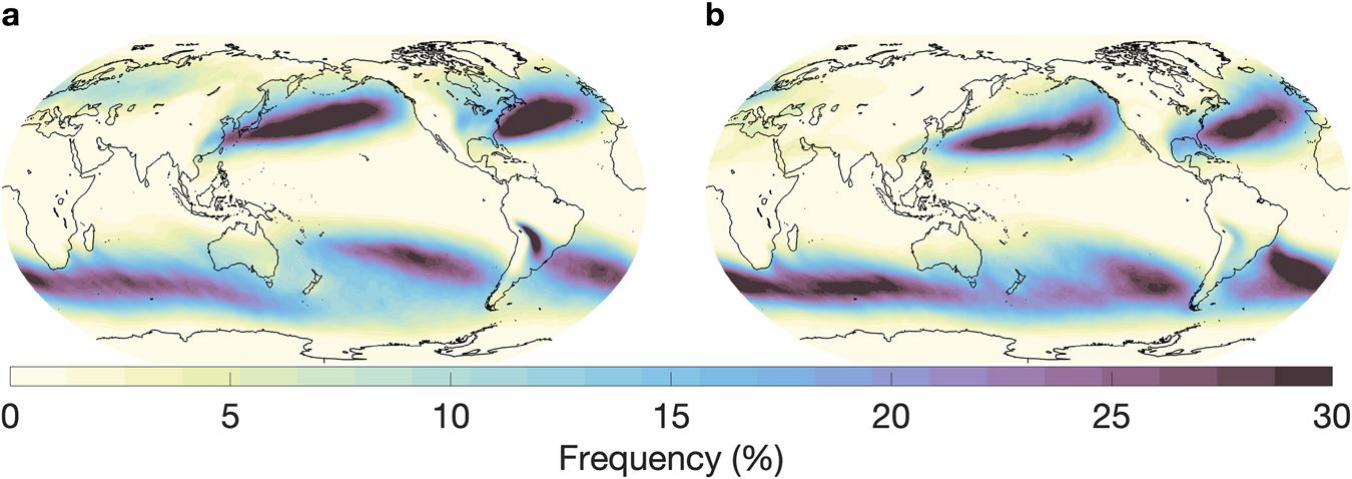
Seasonal global atmospheric river frequencies based on all annotated timesteps for (**a**) June, July, August and (**b**) December, January, February.

**Fig. 6:**
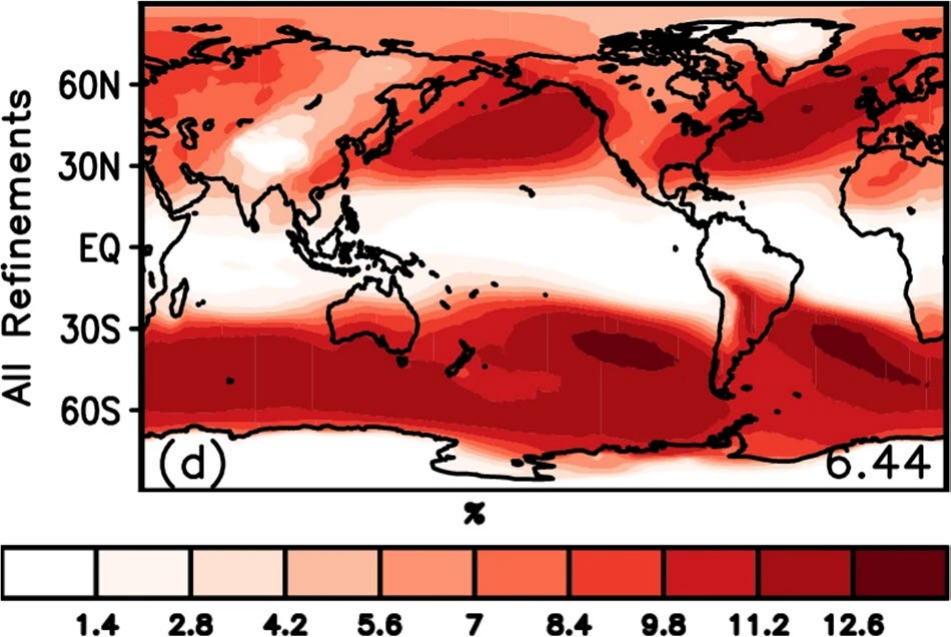
Annual global atmospheric river frequencies detected by the most updated version of tARget (figure directly from Guan and Waliser^27^, Fig. 6d, Open Access, Creative Commons Attribution version 4.0 International License).

In this section, we provide an overview of the hand-labeled extreme weather events and compare them with observations or other published methods for detecting the respective features. We begin by comparing our AR labels to those detected by a widely used, continuously developed algorithm termed *Tracking Atmospheric Rivers Globally as Elongated Targets* (tARget)^25–27^. The most recent update to the tARget algorithm was evaluated on ERA5 for the period 1940-2023, which matches the dataset underlying our labeling campaign, but covers a longer time period^27^. The latest updates to the tARget algorithm refined the detection of ARs in polar regions, tropical regions, and zonal ARs. Fig. 4 displays the global frequency (%) of our AR hand-labels, which can be compared with the results from the most recent study by Guan and Waliser, shown in Fig. 6^27^. Atmospheric river distributions are in good agreement in terms of the well-known zones of high activity, e.g. the Pacific and Atlantic subtropics and mid-latitudes, but there are differences in magnitude. The hand-labeled AR dataset shows higher magnitudes compared to the tARget algorithm; over the north/south Atlantic, north Pacific, and southern Indian oceans, maximum frequencies approach or reach 30% for the hand-labeled dataset as compared to 10-12% with the tARget algorithm. We note that a similar magnitude difference was observed for domain-experts when asked to hand-label ARs, i.e. it is not a specific issue of crowd-sourced labels, but rather expresses the expected discrepancies between human annotators (expert or not) and other detection algorithms, likely due to inherent definition uncertainty^9^. Also, the Atmospheric River Tracking Method Intercomparison Project (ARTMIP), which compared a variety of hand-engineered and learning-based detection algorithms, exhibited similar variability^8,9,28^. In the polar regions, particularly over the Arctic, and over some land areas, such as the Middle East and India, the hand-labeled AR dataset shows lower frequencies when compared to the tARget algorithm. To note, the tARget algorithm’s most recent refinements generated higher frequencies of polar ARs. Even prior to the refinement, the tARget algorithm was unique in its ability to detect ARs over polar regions and over land compared to other algorithms as it uses a location and month specific relative threshold for IVT^27,28^. Seasonal frequencies of AR hand-labels (June, July, and August (JJA); December, January, and February(DJF)) are shown in Fig. 5. A study on the JJA and DJF seasonal AR frequencies in ERA5 has shown higher frequencies of ARs during JJA over the subtropical south Pacific, western north Pacific, and north Indian oceans as compared to DJF frequencies^29^. Our hand-labels well-represent these seasonal differences apart from the lack of AR frequencies over the north Indian Ocean during JJA.

For TCs, we compare our results with two other studies: Sobel *et al*.^30^, and Knapp *et al*.’s International Best Track Archive for Climate Stewardship (IBTrACS) data set^31^. Using ERA5 data from 1980-2016, Sobel *et al*. designed a tropical cyclone genesis index that combines four predictors related to tropical cyclones: potential intensity, vertical wind shear, absolute vorticity at 850 hPa, and column integrated relative humidity^30,32^. The index quantifies the probability that a tropical cyclone will form over a given region. Fig. 8 shows the global frequency (%) of tropical cyclones from our human-labels and Fig. 10 shows the tropical cyclone genesis index. The known areas of high TC activity are consistent across the two maps, including the western and eastern Pacific just north of the equator, the Indian Ocean and western Pacific just south of the equator, and lastly, weaker activity in the western Atlantic north of the equator. Next, we compare our hand-label frequencies of TCs with IBTrACS^31^ which is a comprehensive collection of global tropical cyclone best-track data. Knapp *et al*. generated frequencies of storms per decade from 1945-2007 (Fig. 3 of Knapp *et al*.^31^; not shown in this study). Again, zones of high activity are in strong agreement with our hand-labels although the IBTraCS magnitudes peak at around 1% which is lower than our magnitudes. The Knapp *et al*. frequencies were generated by taking tropical cyclone tracks and expanding them by 1° which corresponds to a distance, or radius, of a half-degree (55 km) around each track point. However, previous studies have found the radius of observed tropical cyclones to be far greater than a half-degree with sizes ranging from 2.7° to well above 10° for the largest cyclones^33,34^. As this dataset segments the entire tropical cyclone shape, our frequencies would be expected to have higher frequencies than those reported in IBTrACS. To demonstrate, Cheng *et al*. show how TC frequencies change when the size of the TC is assumed to be a 1° area grid box around a track compared to 8°; TC annual frequencies maximize at around 1% (in line with IBTrACS) compared to around 15% for those respective grid box sizes (Fig. 11); our TC frequencies fall within those ranges. Examining the seasonal frequencies of our TC hand-labels (Fig. 9) shows higher TC activity in the north Pacific and Atlantic during JJA and higher TC activity over the southern Indian Ocean and south Pacific. These results are in agreement with observed seasonal TC frequencies^30^.

**Fig. 7:**
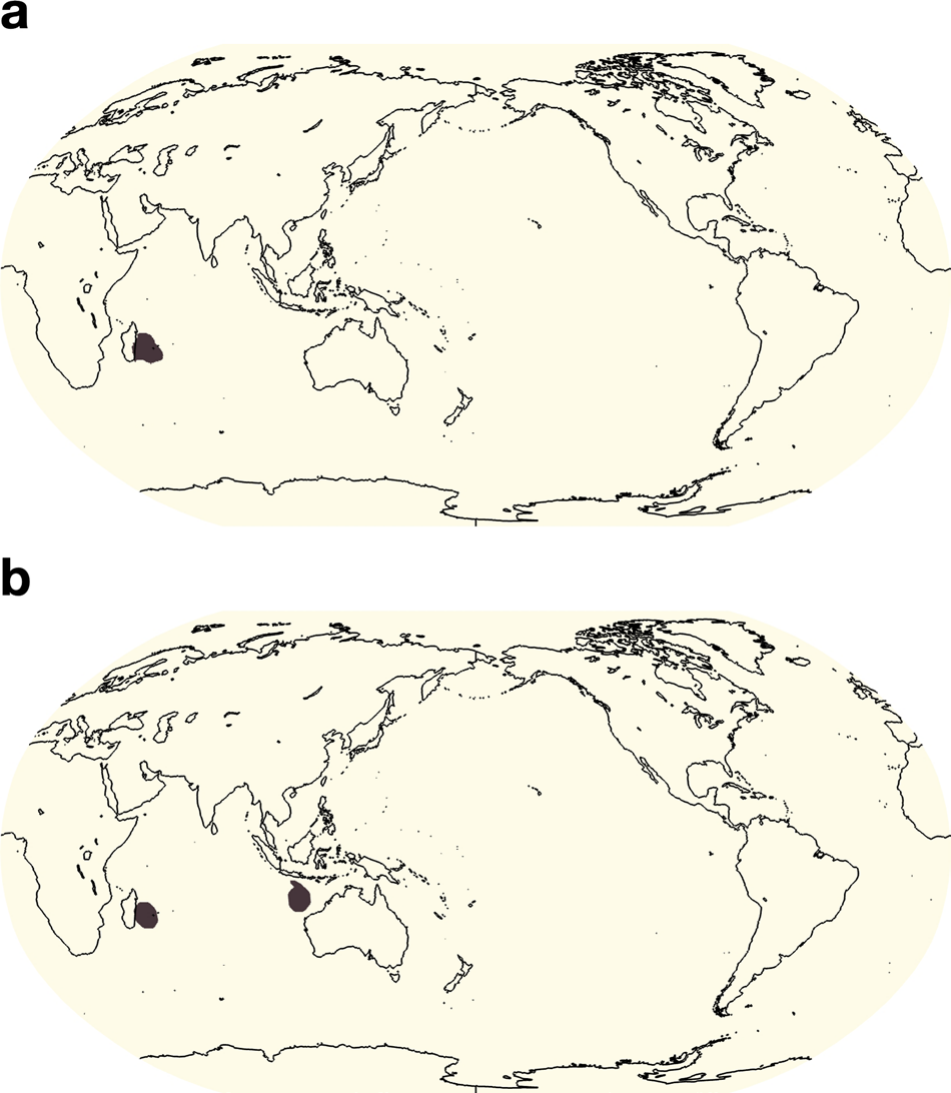
(**a**) and (**b**) show two different annotators’ labels of tropical cyclones for the same timestep. Both annotators find a cyclone off the eastern shore of Madagascar while annotator (**b**) includes another cyclone northwest of Australia.

**Fig. 8:**
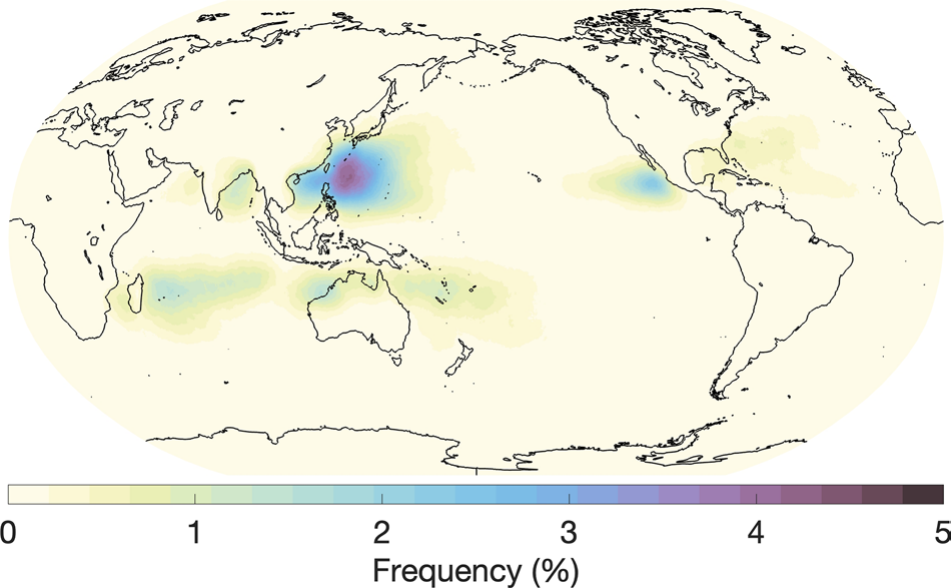
Annual global tropical cyclone frequencies based on all annotated timesteps and all annotators.

**Fig. 9:**
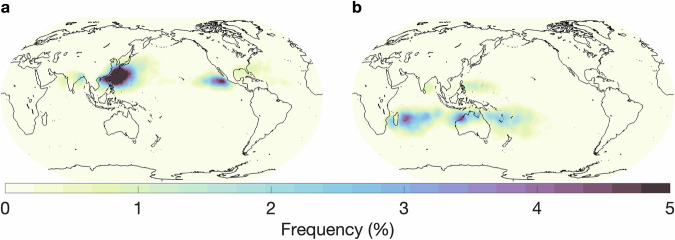
Seasonal global tropical cyclone frequencies based on all annotated timesteps for (**a**) June, July, August and (**b**) December, January, February.

**Fig. 10:**
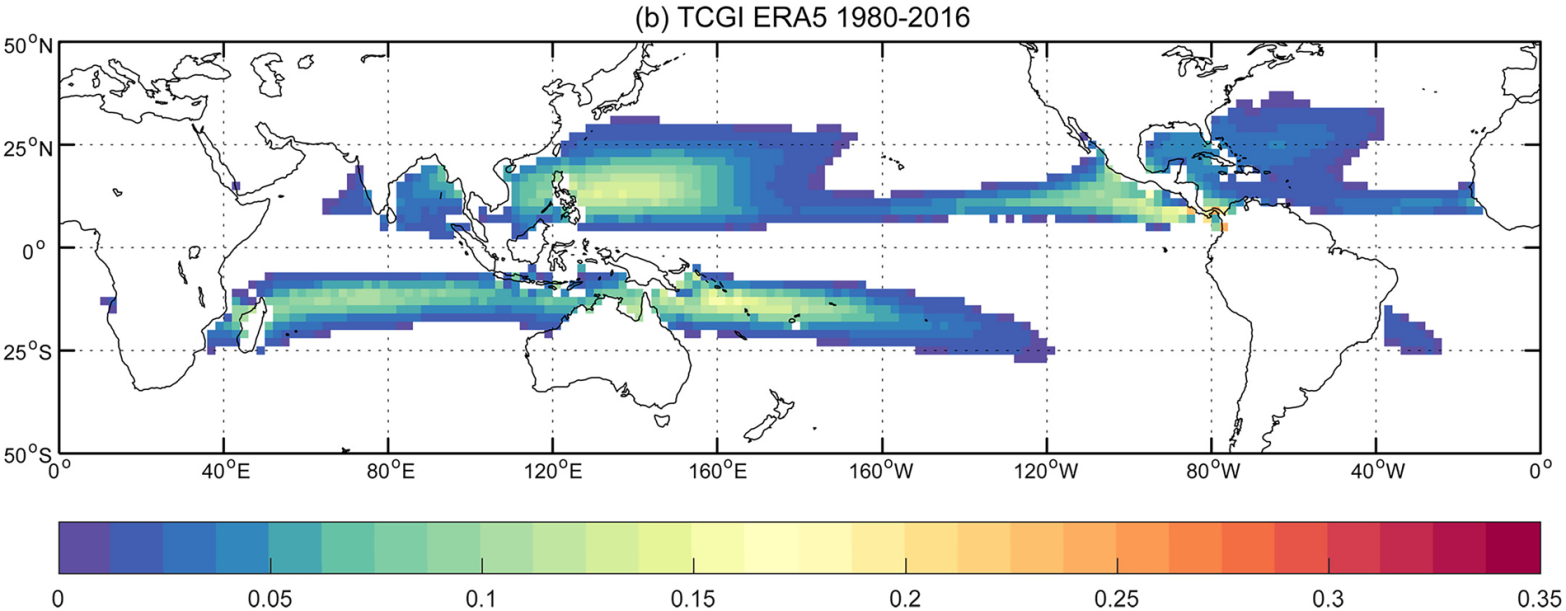
Global tropical cyclone genesis index based on four environmental fields from ERA5 (figure directly from Sobel *et al*.^30^, Fig. 3b, Open Access, Creative Commons Attribution version 4.0 International License).

**Fig. 11:**
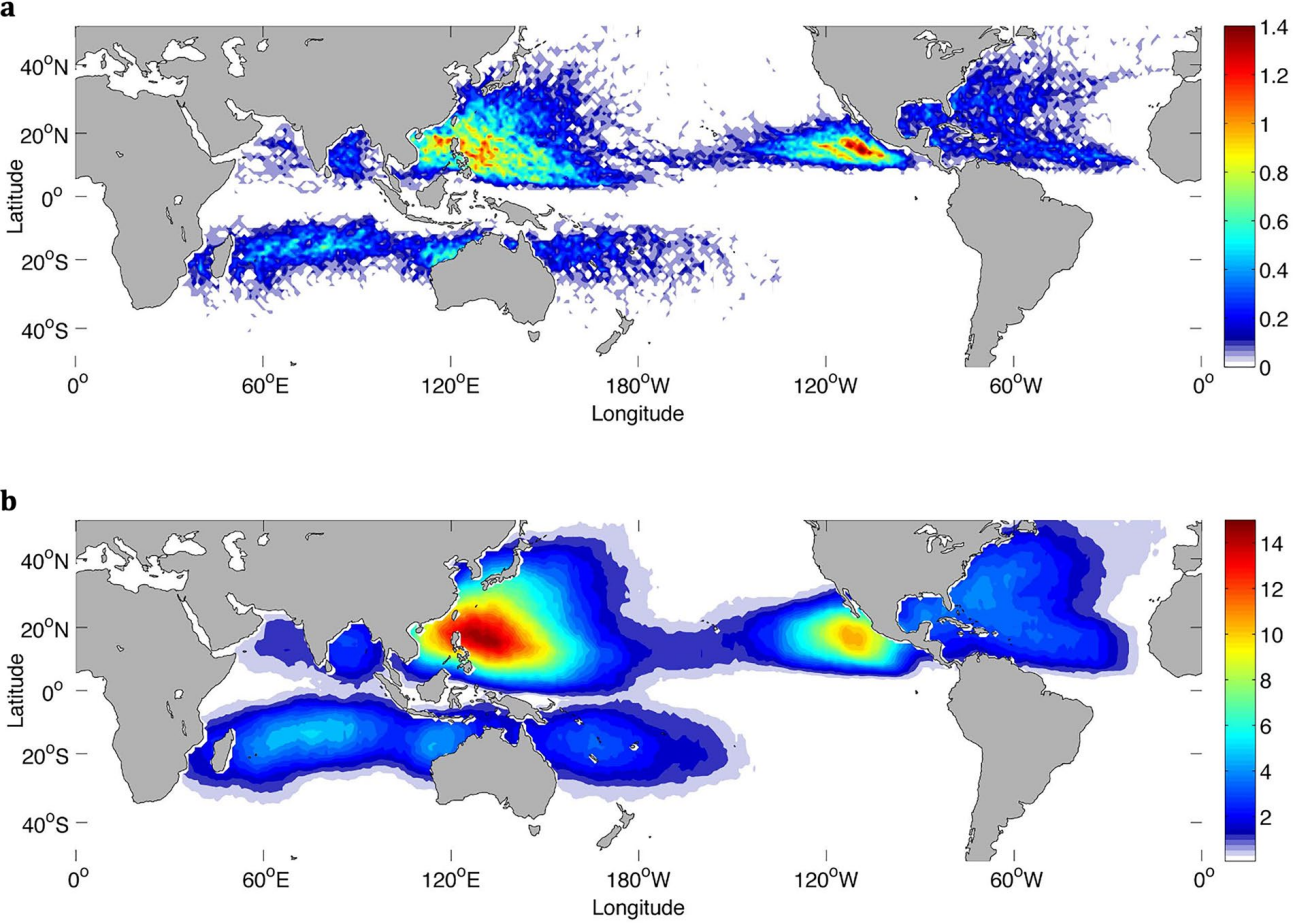
Global tropical cyclone frequencies based on observations assuming a 1° grid box around the track center (**a**) and a 8° grid box around the track center (**b**) (figure directly from Cheng *et al*.^43^, Fig. 7, Open Access, Creative Commons Attribution version 3.0 License).

**Fig. 12:**
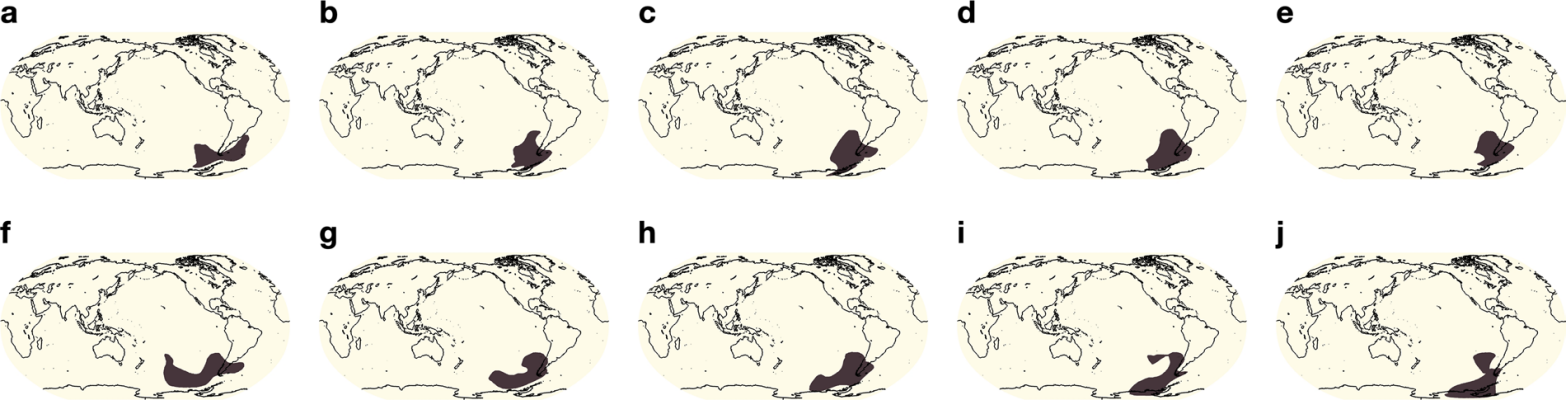
(**a**–**j**) shows the evolution of a blocking event over 10 consecutive days off the southern coast of South America.

**Fig. 13:**
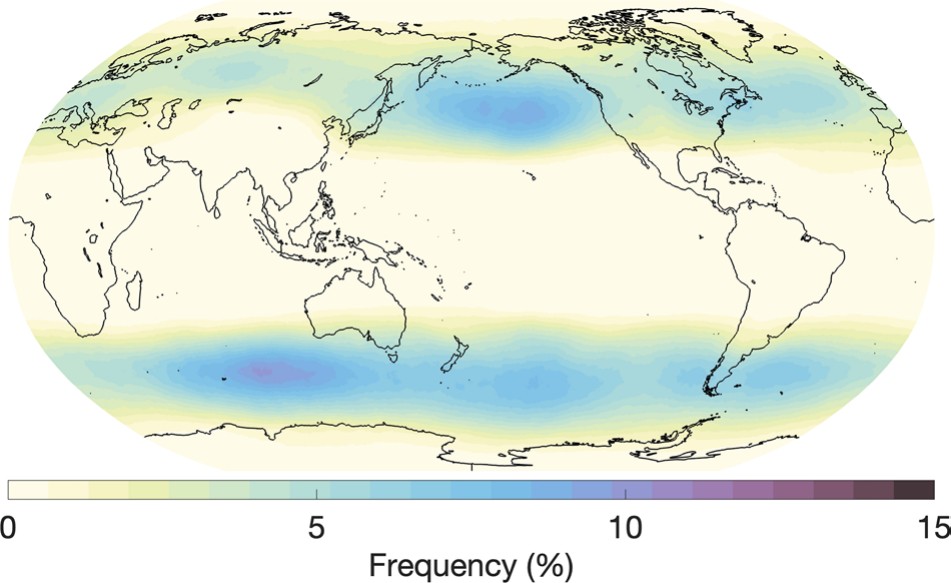
Annual global atmospheric blocking frequencies based on all annotated timesteps and all annotators.

**Fig. 14:**
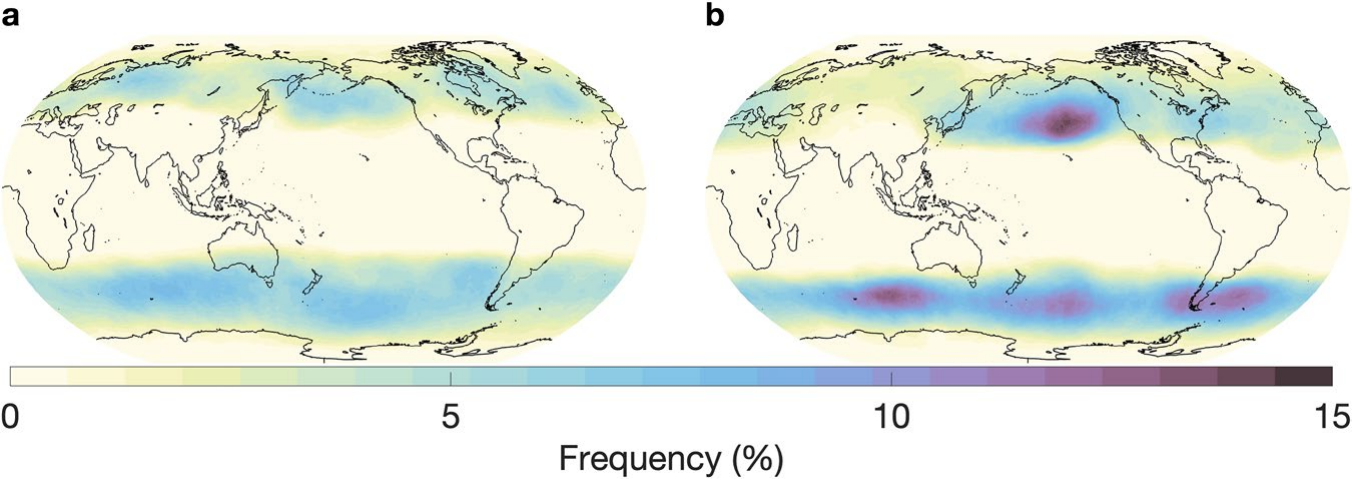
Seasonal global atmospheric blocking frequencies based on all annotated timesteps and all annotators for (**a**) June, July, August and (**b**) December, January, February.

**Fig. 15:**
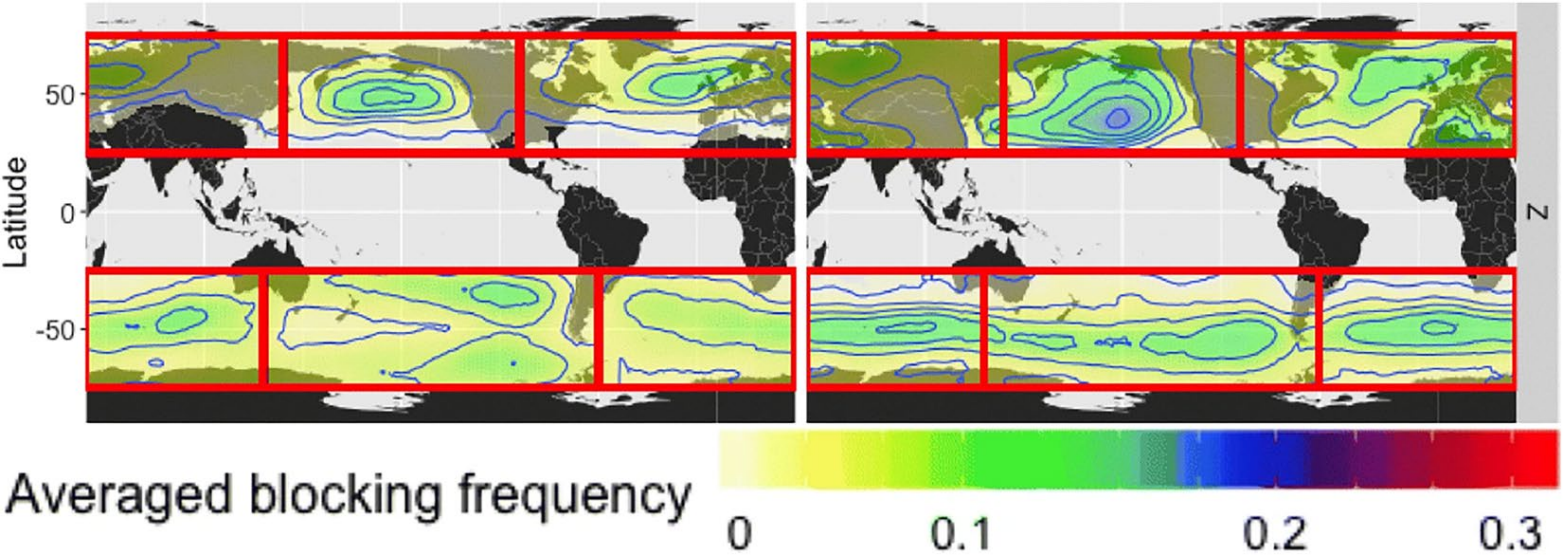
Seasonal global atmospheric blocking frequencies based on the geopotential height anomaly detection algorithm by Pinheiro *et al*. Left - June, July, August; right - December, January, February. (figure directly from Pinheiro *et al*.^6^, Fig. 6 center row, Open Access, Creative Commons Attribution version 4.0 International License).

Lastly, we compare the atmospheric blocking results to a study by Pinheiro *et al*. where they utilized the European Centre for Medium-Range Weather Forecasts’ reanalysis product, known as ERA-Interim, for the years 1979-2018^6^. Among the different versions of their algorithm, we compare to the one that focuses on the 500 hPa geopotential height anomaly, the same field shown to the crowd-labelers in our study. As criteria for blocking, that version requires that a grid point must exceed 100 m (for the geopotential height anomaly at 500 hPa) for 10 consecutive days. There is no universally accepted definition for blocking events and other height anomalies and durations have also been used. The global frequency of atmospheric blocking events for our study is shown in Fig. 13 (annual) and Fig. 14 (seasonal). The results from Pinheiro *et al*. are depicted in Fig. 15. Overall, both the magnitude and distribution of atmospheric blocking events show good agreement for both seasons, with blocking occurring predominantly in the north and south Pacific Ocean, south Indian Ocean, and north and south Atlantic. Over land, significant areas of blocking occur over northeastern areas of North America and northwestern and eastern areas of Europe. The DJF months show stronger blocking in the north Pacific and southern storm tracks compared to JJA for both studies. Maximum frequencies are around 15% (especially during DJF), with a prominent band of 5–10% around 50° north and south of the equator.

Overall summary statistics suggest that crowd-labelers were able to map ARs, TCs, and atmospheric blocking events well enough to reproduce the frequencies and distributions found in existing studies. There are some differences in frequency magnitude, for which we have provided plausible explanations. While the dataset produced provides labels of extreme events ready for the training of deep learning models, there are some present limitations; the labels purely denote areas that labelers defined for extreme events without *(i)* further details such as the centroid location, direction, and tags for landfalling and *(ii)* underlying climate/weather characteristics such as precipitation, wind speed, temperature, and humidity associated with each label. These details and characteristics can be determined with post-processing of labels and integration of reanalysis data for the corresponding time steps.

## Data Records

Our efforts to make the new data set accessible include source code for efficient handling of the data. We provide NetCDF files for structured file access^24^. The labels for ARs, TCs, and blocking events are packaged with the following variables: time, label, class, annotator, longitude, and latitude. The “label" variable is 3-dimensional and contains annotator, latitude and longitude dimensions (blocking events are 4-dimensional with the addition of a time dimension). The “class" dimension denotes the event type. The “annotator" dimension denotes two separate annotators. Latitude and longitude are arrays of 721 and 1440 length respectively for quarter-degree intervals. ARs and TCs are packaged in single timesteps. The labels for atmospheric blocking are packaged in sequences of 10 consecutive days. Each file name denotes the timestep (for blocking the first timestep). Labeled and background grid cells are denoted as 1 and 0 respectively. The labels for ARs, TCs, and blocking events are separated by directory, in the manner described above, and are available at Harvard Dataverse^35^. The data suffixed by “raw" are the raw dataset before the area threshold filters were applied and the data suffixed by “cleaned" are the dataset after filters were applied.

## Technical Validation

**Fig. 16:**
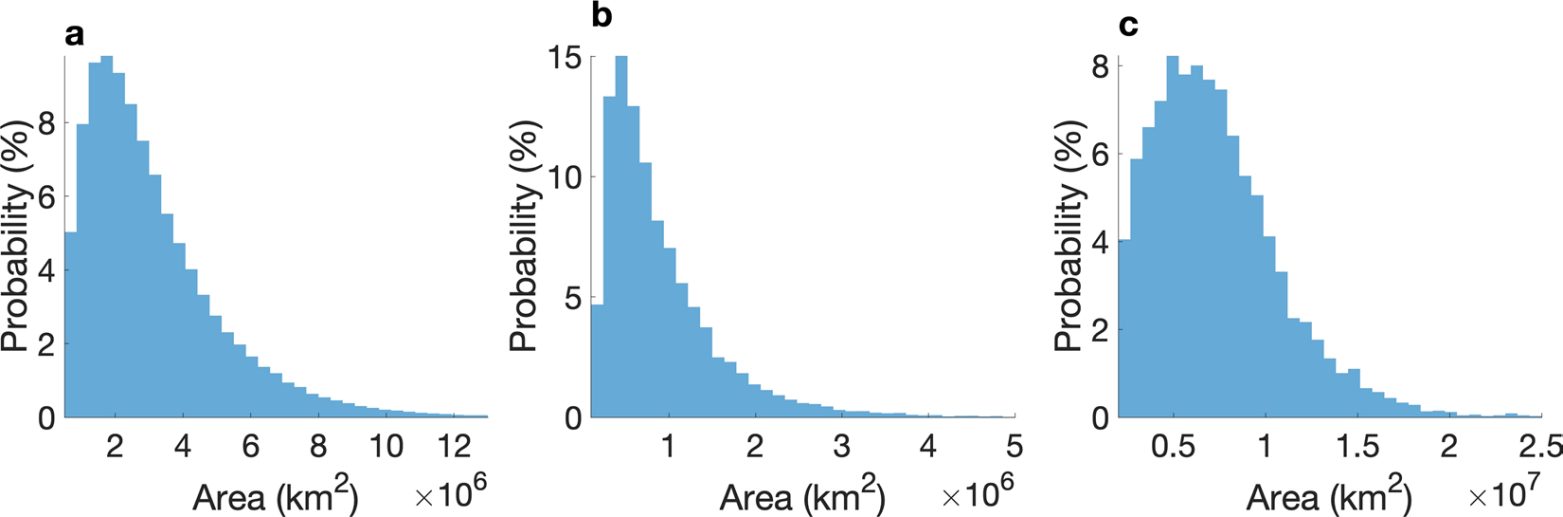
Probability distributions of area of events for (**a**) ARs, (**b**) TCs, and (**c**) blocking events.

For all three event types, validation testing was done to minimize the risk of annotation errors in the final dataset although we cannot completely rule out human mislabels and errors. ARs were tested to identify and remove blank timesteps (ARs should exist in every timestep^15^) and duplicate timesteps. TCs were tested to identify and remove duplicates for all timesteps in which a TC exists. Blocking events were tested to identify and remove duplicates if an entire file (10 consecutive days) had matching duplicates with another file. Additionally, we set size thresholds for each event type to remove errant markings and unrealistically small events made by the human labelers. ARs under 250,000 sq km, TCs under 100,000 sq km, and blocking events under 2,000,000 sq km were removed. Fig. 16 shows probability distributions for all three events. The minimum length of ARs with available detection algorithms are typically 2000 km with widths at least half as small^8^. Median length and width of a commonly used algorithm are 3665 km and 564 km respectively^25^ and our area distributions match well with another AR area evaluation^36^. TCs typically have a minimum outer radius of around 200 km which corresponds to an area of around 125,663 sq km assuming a circular shape^37^ and size classifications for tropical cyclones are defined as “very small” if their radius of outermost closed isobar (ROCI) is less than 2°. Blocking events defined by other detection algorithms set minimum sizes to 2,000,000 km sq^38,39^. Areas of each event were calculated using SciPy’s label function with connected areas defined with 8-connections (i.e. connection is considered if any of the 8 surrounding pixels is also annotated)^40^. We also handled cases where labels were considered connected at the edges of the data longitudinally (e.g. an AR beginning around 350° E and ending around 10° E). The mean Intersection-over-Union metric was calculated to compare our crowd-labelers against each other for ARs, TCs, and blocking events. We find values of 0.42, 0.22, and 0.35 respectively; these numbers are comparable to mean Intersection-over-Union values for domain-expert against domain-expert labels for ARs and TCs (0.34 for ARs and 0.26 for TCs)^9^.

## Usage Notes

For many analysis tasks it may be helpful to combine all timesteps for a given event type, for instance when looking for seasonal statistics and patterns. To combine multiple (or all) timesteps together or performing seasonal analysis, we recommend the use of NetCDF tools^41^ or Climate Data Operators^42^.

## Data Availability

The code used to process ERA5 data from NetCDF format into an appropriate format for webKnossos along with uploading to the web interface is available. Although our dataset is provided in already packaged form, pre-processing code to download webKnossos annotations and package them into the NetCDF format is also available. These are both available on GitHub at the following URL: https://github.com/andregraubner/ClimateNetLarge .
